# Whole-Genome Sequences of Two Clinical Isolates of Extensively Drug-Resistant *Mycobacterium tuberculosis* from Zunyi, China

**DOI:** 10.1128/genomeA.00910-14

**Published:** 2014-09-18

**Authors:** Ling Chen, Derek T. Zhang, Jianyong Zhang, Yan A. Su, Hong Zhang

**Affiliations:** a Department of Respiratory Medicine, Affiliated Hospital of Zunyi Medical College, Zunyi, China; b Z-BioMed, Inc., Rockville, Maryland, USA

## Abstract

Before 2013, 92 countries reported extensively drug-resistant *Mycobacterium tuberculosis* cases to the WHO. Here, we announce the genome sequences of two clinical isolates of extensively drug-resistant tuberculosis (XDR-TB) from Zunyi, China. The genome sequences are composed of 4,411,507 bp and 4,411,515 bp with 2,210 and 2,071 variants, respectively, when compared to the H37Rv genome.

## GENOME ANNOUNCEMENT

Tuberculosis (TB) caused about 1.3 million deaths worldwide in 2012, and about 3.6% of newly diagnosed TB cases along with 20% of previously treated TB cases in the world were multidrug-resistant tuberculosis (MDR-TB) (1). By the end of 2012, 92 countries reported extensively drug-resistant TB (XDR-TB) cases to WHO and an estimated 9.6% of MDR-TB cases were XDR-TB (1). The continued spread of MDR/XDR-TB is a serious threat to all nations of the world with the potential of making health care infrastructures useless in developing countries, such as China. Results from national surveys of drug-resistant TB in China confirmed that China faces serious challenges in combating MDR/XDR-TB, and primary transmission accounts for most of the MDR/XDR-TB cases (2, 3). Previously, we reported that the prevalence of MDR/XDR-TB in Zunyi, Guizhou Province, was higher than China’s national average prevalence (4).

Several XDR-TB genomes from different countries, including China, have been sequenced (5–9), but none of them is specifically from Zunyi, Guizhou Province. To better understand the genetic mechanism underlining the extensive drug-resistance of *M. tuberculosis* strains and to identify new mutations in known and new genes associated with XDR, it is necessary to sequence the whole-genomes of clinical isolates of XDR-TB. Here, we announce the draft whole-genome sequences of two XDR-TB strains, ZMC13-264 and ZMC13-88, isolated in 2013 from TB patients in Zunyi, Guizhou Province of China.

Whole-genome sequencing was performed by using an Ion Torrent sequencing platform (Life Technologies). The genomic DNA samples of ZMC13-264 and ZMC13-88 were sequenced to about 290× coverage. The process generated 3,234,924 and 3,435,034 total reads, 632,975,164 and 653,240,179 total bases, with mean read lengths of 195 bp and 190 bp for ZMC13-264 and ZMC13-88, respectively. The draft whole-genome sequences for ZMC13-264 and ZMC13-88 are composed of 4,411,507 bp and 4,411,515 bp, containing 2,210 and 2,071 variants, respectively. We obtained the numbers of variants by comparing the genomes to the *M. tuberculosis* H37Rv complete genome (GenBank accession no. NC_000962.3). The G+C contents for both genomes are almost the same (65.60%). The assembled sequences were annotated by using the Rapid Annotation of microbial genomes using Subsystems Technology (RAST) (10), which revealed 3,914 and 3,917 protein-coding genes, along with 50 and 48 RNA-coding genes for ZMC13-246 and ZMC13-88, respectively.

The announcement of whole-genome sequences of two XDR *M. tuberculosis* strains, ZMC13-264 and ZMC13-88, isolated from patients in Zunyi, Guizhou Province of China, will provide a foundation for comprehensive comparison with XDR-TB genomes from different countries and regions of the world (5–9), as well as the standard reference H37Rv genome (11). The comparative genomic analysis of these genome sequences will facilitate the identification of new mutations in known and/or new genes associated with XDR-TB and give us more insights into the genetic and epidemiology variations occurring in XDR-TB strains circulating in Zunyi, Guizhou Province of China.

### Nucleotide sequence accession numbers.

The whole-genome sequences of two XDR-TB strains from Zunyi, China, have been deposited in the GenBank database under accession numbers CP009100 and CP009101.
